# Gender differences in artificial intelligence: the role of artificial intelligence anxiety

**DOI:** 10.3389/fpsyg.2025.1559457

**Published:** 2025-05-06

**Authors:** Claudia Russo, Luciano Romano, Davide Clemente, Leonardo Iacovone, Thomas Edward Gladwin, Angelo Panno

**Affiliations:** ^1^Experimental and Applied Psychology Laboratory, Department of Health and Life Sciences, Università Europea di Roma, Rome, Italy; ^2^Department of Psychology, University of Chieti, Chieti, Italy; ^3^Institute for Globally Distributed Open Research and Education (IGDORE), London, United Kingdom

**Keywords:** artificial intelligence, anxiety, AI anxiety, attitudes, gender differences

## Abstract

**Introduction:**

Artificial intelligence (AI) is having a significant impact on people's lives. Despite the benefits associated with this technological advancement, there may be gender-related inequalities in accessing and using AI systems. The present study aimed to test gender differences in factors likely to influence AI adoption, in particular, the moderating role of gender in the relationship between AI anxiety and positive attitudes toward AI.

**Method:**

Participants were 335 adults (52.2% women; mean age = 29.96, SD = 13.88) who filled in an online self-report anonymous questionnaire. To test the hypotheses, both a MANOVA and a moderation model were adopted.

**Results:**

Results revealed significant gender differences in AI adoption dimensions, with women reporting higher AI anxiety, lower positive attitudes toward AI, lower use of AI, and lower perceived knowledge of AI. A significant negative relationship was found between AI anxiety and positive attitudes toward AI. An interaction between gender and AI anxiety was found: At low levels of anxiety, women showed lower levels of positive attitudes toward AI than men, while at high levels of AI anxiety, gender differences were less evident.

**Discussion:**

These findings suggest that AI anxiety works as a “gender differences leveler.” The present study contributes to expanding knowledge about gender differences in technology, which will underpin practical interventions for reducing the gender digital gap. Limitations and future research directions are discussed.

## 1 Introduction

The current state of Artificial Intelligence (AI) represents the culmination of decades of studies on machine learning, neural networks, and data analysis; these research efforts have been made to enable machines to simulate human-like actions and decision-making processes (McCarthy et al., 2006; Kaplan and Haenlein, 2019; Park and Woo, 2022). AI can be seen as one of the most transformative technological advances of the 21st century, significantly impacting various dimensions of human life (Makridakis, 2017). From healthcare to entertainment, from industry to education, AI is shaping new ways to work, communicate, and solve both daily and complex problems (De Felice et al., 2022).

Given its transformative impact, AI cannot be only considered from a technological perspective since it also has significant societal implications. Its rapid ascent raises questions about ethics, equity, and accessibility. Among them, AI can amplify existing biases if the data used to train algorithms reflect inequalities (Ferreira et al., 2021). Moreover, AI-driven transformation may negatively affect gender equality. On the one hand, evidence suggests that the automation of repetitive and routine jobs, which are mainly held by women (Delgado Cadena, 2020), could exacerbate existing economic inequalities (Vorobeva et al., 2022). On the other hand, the underrepresentation of women in technology-related roles and the gender digital skills gap could limit women's ability to benefit from the opportunities offered by advances in AI (Davaki, 2018).

However, as noted by Schepman and Rodway (2023), contrary to other kinds of technologies (e.g., tablets), people have fewer choices about whether or not to directly or indirectly interact with AI systems because these are increasingly becoming pervasive in everyday life, and their regulation mainly depends on governments and large corporations (Chen and Wen, 2021). Both ethical concerns and the feeling of a lack of control behind AI development and usage can lead people to feel more insecure and uncertain about AI, introducing a novel form of anxiety, labeled AI anxiety, which refers to an affective state characterized by fear and apprehension toward AI (Wang and Wang, 2022) that may significantly impact attitudes toward these systems (see, for example, Carrasco et al., 2019; Stephanidis et al., 2019; Triberti et al., 2021).

Considering this evidence, we believe it is noteworthy to investigate gender differences related to AI adoption and to examine the interplay between AI anxiety and gender in shaping attitudes toward AI, with the ultimate aim of fostering inclusive technological integration into society. Thus, the present study aims to test gender differences in mean levels of AI anxiety, positive attitudes toward AI, perceived knowledge of AI functioning, and AI use. Furthermore, we aimed to investigate the association between AI anxiety and positive attitudes toward AI, considering the role of gender in moderating this relationship.

### 1.1 Conceptual overview

Attitudes toward AI might be influenced by both psychological dispositions and socio-demographic characteristics. Among these, an increasingly noteworthy research topic strictly related to the gender digital gap is the impact of gender differences on AI adoption.

Indeed, institutions and policymakers have increasingly focused on gender inequalities in the digital context, aiming to understand these disparities better and identify effective strategies for intervention. According to Davaki (2018), women's access to and use of digital technologies is significantly lower than men's, and women are less likely to own and regularly use devices with internet access. These disparities can arise from various economic, cultural, and social barriers (EIGE, 2016). Among these barriers, it is worth noting that women and girls tend to be less socialized in STEM disciplines and technologies. Only 9.6% of women in the EU pursue ICT-related academic studies, compared to 30.6% of men. This disparity is also reflected in the career paths: of the small number of female ICT graduates, only 4% are actually working in the sector (European Commission, 2013). Although there is insufficient evidence of inherent biological differences between girls' and boys' abilities in STEM subjects (Wang and Degol, 2017; Alam, 2022), there are different stereotypes about these abilities (Schuster and Martiny, 2017). Teaching materials present gender biases, and teachers often use different motivational approaches for girls and boys (Davaki, 2018). Parents reinforce gender stereotypes by encouraging or discouraging boys and girls from pursuing a career in STEM disciplines (Ertl et al., 2017). Finally, girls' career choices in STEM, especially ICT, are also not facilitated by the lack of female role models in science and technology (European Parliament, 2012). These gender-biased socialization processes may result in gender differences in attitudes toward technology and its use. Consistently, several empirical studies showed a trend in which, compared to men and boys, women and girls usually hold more negative attitudes toward technology and technology use and are less engaged in technology-related activities (Sáinz and López-Sáez, 2010; Yau and Cheng, 2012; Ardies et al., 2015). Moreover, as Méndez-Suárez et al. (2023) noticed, women tend to report worse perceptions of devices based on AI, considering them as less socially desirable than men (Kuo et al., 2009; Schermerhorn et al., 2008).

Considering this evidence, we hypothesize that:

*H1: Women will report higher levels of AI anxiety, lower levels of positive attitudes toward AI, lower levels of perceived AI knowledge, and lower levels of AI use compared to men*.

Other than socio-demographic characteristics, psychological characteristics might also have a significant role in shaping attitudes toward AI. Consistently, attitudes toward AI include affective, cognitive, and behavioral components, driven by the representations that an individual holds of AI (Park and Woo, 2022). As with attitudes in general (Breckler, 1984), these three components are closely interrelated, and they uniquely contribute to shaping an overall attitude toward AI as a social object. This attitude might assume a negative or positive valence (Eagly and Chaiken, 2007). Positive attitudes toward AI include several facets: the perception of utility, such as the possibility of gaining economic opportunities or reaching improved working performance; the desirability of AI usage at work or in daily activities; and the positive emotions associated with these systems, such as excitement, or being impressed in the face of this technological advancement (Schepman and Rodway, 2023). Shaping positive attitudes toward AI is critical to fostering its acceptance and integration into society; however, the formation of such positive attitudes is a complex process. Attitudes are shaped by several psychological factors, such as beliefs, emotions, prior experience, and knowledge of the social object (Breckler, 1984; Eagly and Chaiken, 2007; Park and Woo, 2022; Asio and Gadia, 2024). Among these factors, AI anxiety might play a pivotal role.

Recently developed by Wang and Wang (2022), the construct of AI anxiety refers to a “new form” of technology anxiety, which can be defined as an affective response of fear and anxiety that inhibits individuals from interacting with AI systems. It is a form of state anxiety that may vary in response to changing conditions (Bolliger and Halupa, 2012). According to the authors, AI anxiety can be considered a psychological factor that leads to behavioral intentions, potentially influencing attitudes (Wang and Wang, 2022).

Although substantial findings are still lacking in this direction, recent evidence showed a significant association between AI anxiety and attitudes toward AI (Kaya et al., 2024). Moreover, previous research in the Information Technology (IT) context highlighted that higher levels of technology anxiety are associated with greater levels of frustration during technology integration processes, leading people not to support technology change (Henderson and Corry, 2021). Other evidence pointed out that technology anxiety is a significant predictor of resistance to technology, working as a barrier to an individual's involvement in approaching new technologies (Thatcher and Perrewe, 2002; Troisi et al., 2022). Indeed, anxiety can significantly affect the perceived usefulness and the perceived ease of use of technology, both considered by the Technology Acceptance Model (TAM; Davis, 1989) as the main antecedents of behavioral intention in technology use (Meuter et al., 2003; Aggelidis and Chatzoglou, 2009; Chen and Tseng, 2012; Alrajawy et al., 2018). Several previous findings showed a significant and negative relationship between computer anxiety and positive attitudes toward the use of computers and the Internet (e.g., Durndell et al., 2000; Hong and Koh, 2002; Popovich et al., 2008; Korobili et al., 2010).

Based on these considerations, we hypothesized that:

*H2: AI anxiety will be negatively associated with positive attitudes toward AI*.

Moreover, drawing on the perspective of AI-related gender equality, it is worth considering evidence that gender differences may influence the relationship between anxiety and attitudes toward technologies (Cai et al., 2017). Consistently, specifically focusing on AI, a recent study highlighted that men are more likely to use AI systems than women, while women are more likely to perceive AI systems as being more complex to learn and less useful in addressing their needs (Ofosu-Ampong, 2023). Moreover, other preliminary findings showed that high school students hold different levels of positive attitudes toward AI based on their gender, with girls reporting significantly lower levels of overall positive attitude toward AI (Beig and Qasim, 2023), although another study did not find a significant effect of gender on attitudes toward AI (Kaya et al., 2024). Finally, Borwein et al. (2024) pointed out that women reported lower levels of knowledge of AI systems and technologies and higher levels of concern regarding the use of AI in working contexts than their men colleagues. Sobieraj and Krämer (2020) argued that these gender differences result from socialization processes based on different gender roles for men and women. Beliefs about gender roles reflect culturally shared expectations about how women and men are supposed to behave in society (e.g., Prentice and Carranza, 2002). These beliefs function as cognitive schemas or stereotypes, shaping how individuals mentally represent and categorize women and men (e.g., Sobieraj and Krämer, 2020). Stereotypical gender role expectations typically attribute traits like warmth, care, and compliance to women and traits like rational, ambitious, assertive, and STEM skilled to men (e.g., Cuddy et al., 2008; Haines et al., 2016). One of the first studies related to the topic found that differences between women and men in terms of computer skills and usage could be traced back to distinct socialization processes, with technology that has traditionally been framed as more congruent with male than female gender roles (Gefen and Straub, 1997). More recently, Morahan-Martin and Schumacher (2007) argued that computers and technology have come to represent a symbol of masculinity, contributing to women's disengagement by reinforcing a sense of exclusion from a space culturally coded as “not for them.”

Drawing on this perspective, it might be possible that women's relationship with technology—including AI—is characterized by a pre-existing sense of alienation or perceived inadequacy stemming from long-standing socialization processes and gender role expectations. As a result, women's negative attitudes toward AI may be shaped more by structural distancing from technology-related domains. In contrast, men's engagement with AI may be less constrained by such gendered barriers and, therefore, more susceptible to variation depending on their emotional experiences, such as anxiety. In this scenario, at low levels of AI anxiety, women may show low levels of positive attitudes toward AI, whereas at high levels of AI anxiety, both women and men will show less positive attitudes toward AI.

Thus, we hypothesized that:

*H3: The relationship between AI anxiety and positive attitudes toward AI would vary as a function of gender*.

In particular, it was expected that gender moderates the negative impact of AI anxiety on positive attitudes, with women showing lower positive attitudes even at low levels of anxiety.

## 2 Method

### 2.1 Participants and procedure

A community sample of 335 adults and young adults (women 52.2%) agreed to take voluntarily part in the study. Aged ranged between 18 and 79 years (*M* = 29.96; SD = 13.88). Most of the participants were Italian (98.2%), single or unmarried (73.4%), and held at least a high school diploma (46.3%). See Table 1 for more details about socio-demographic information.

**Table 1:** Sociodemographic/informational table.

**Variables**	***M***	**(SD)**
Age	29.96	13.88
**Gender**		
Women	175	52.2
Men	160	47.8
Non-binary	0	0
**Nationality**		
Italian	329	98.2%
Romanian	2	0.6
Anglo-Italian	1	0.3
Afghanistan	1	0.3
Cypriot	1	0.3
Mexican	1	0.3
**Educational qualification**		
High school diploma	155	46.3
Bachelor degree	91	27.2
Master's degree	61	18.2
Master	14	4.2
Middle school diploma	9	2.7
PhD or other degree	5	1.5
**Marital status**		
Single	246	73.4
Married or civilly united	53	15.8
Cohabitant	24	7.2
Separated	5	1.5
Divorced	4	1.2
Widower	3	0.9
**Employment status**		
Nonworker	163	48.7
Full-time job	101	30.1
Part-time job	71	21.2

M, mean; SD, standard deviation; N, number; %, percentage.

The present study adopted a cross-sectional design and convenience sampling technique. Participants were informed about the main aims of the study and were assured that participation was entirely voluntary. Participants who gave informed consent filled in an anonymous self-report online questionnaire. Anonymity and confidentiality standards were ensured at every data collection point. The research protocol follows the Declaration of Helsinki of 1964 and its latest versions. The research has been approved by the Ethical Committee of Università Europea di Roma (prot. n. 01/2025).

### 2.2 Measures

#### 2.2.1 AI anxiety

To assess participants' anxiety about AI, we used the AI Anxiety Scale (Wang and Wang, 2022). The scale is composed of 21 items that were translated into Italian using a back-translation method. An example of an item was: “Learning how an AI technique/product works makes me anxious.” The response scale ranged from 1 (“completely disagree”) to 7 (“completely agree”). Cronbach's alpha and McDonald's omega were both 0.94.

#### 2.2.2 Positive attitudes toward AI

To measure participants' positive attitudes toward AI, we used the 12 items of the positive dimension of the General Attitudes Toward Artificial Intelligence Scale (GAAIS) (Schepman and Rodway, 2023). The items were translated into Italian using a back-translation method. An example of an item was: “I am interested in using artificially intelligent systems in my daily life.” The response scale ranged from 1 (“strongly disagree”) to 5 (“strongly agree”). Cronbach's alpha was found to be 0.89, and McDonald's omega was 0.90.

#### 2.2.3 AI systems and technologies use

To evaluate the use of AI systems and technologies, we utilized a single item adapted from Park and Woo (2022). The item was back-translated into Italian. Specifically, the item was: “Have you ever used technologies or systems that make use of artificial intelligence?”, the response scale ranged from 1 (“never”) to 7 (“often”).

#### 2.2.4 Perceived knowledge of AI systems and technologies

To assess prior knowledge of systems and technologies based on AI, a single *ad hoc* item was specifically developed for this study, namely: “How much do you think you are informed about the functioning of artificial intelligence technologies and/or systems?”. The response scale ranged from 1 (“not informed”) to 7 (“fully informed”).

#### 2.2.5 Socio-demographic variables

As regards the socio-demographic variables, we collected information on participants' age, gender (nonbinary, women, men), educational qualification (high school diploma, university degree or higher, etc.), marital status (single, in a relationship, etc.), employment status (nonworker, full-time job, part-time job), and nationality (Italian, other).

### 2.3 Analysis plan

Preliminarily, we described the study's variables in terms of means, standard deviations, range, skewness, and kurtosis. Since all variables exhibited skewness and kurtosis values below |2|, we assumed a normal distribution. Consequently, Pearson's correlation analysis was employed to assess associations between the variables.

To test our hypotheses, we first adopted the MANOVA, including gender (coded as 1 = men; 2 = women) as a factor. Then, we used PROCESS macro for SPSS v. 4.2 (Hayes, 2018), adopting model 1 to test the moderating role of gender in the relationship between AI anxiety and positive attitudes toward AI. Age, prior use of AI, and perceived knowledge of AI functioning were included as covariates in the model. In case of significant interaction, results were plotted using Interaction! v. 1.7 (Soper, 2013) to examine whether and how the relationship mentioned above varies as a function of gender.

## 3 Results

### 3.1 Descriptive statistics

Descriptive statistics are reported in Table 2.

**Table 2:** Descriptive statistics and Pearson's correlation analysis.

**Variables**	***M***	**SD**	**Range**	**SK**	**KU**	**2**	**3**	**4**
1. AI anxiety	3.78	1.18	1.00–7.00	0.19	−0.40	−0.43^**^	−0.35^**^	−0.38^**^
2. Positive attitudes toward AI	3.17	0.78	1.00–5.00	−0.16	0.39	–	0.45^**^	0.32^**^
3. Use of AI	3.77	1.99	1.00–7.00	0.08	−1.15		–	0.54^**^
4. Perceived knowledge of AI functioning	3.56	1.63	1.00–7.00	0.33	−0.58			–

SK, skewness; KU, kurtosis. ^**^p < 0.01.

Results from the correlation matrix in Table 2 reveal that AI anxiety is significantly and negatively associated with positive attitude toward AI, prior use of AI, and perceived knowledge of AI functioning. Positive attitude toward AI is significantly and positively associated with both prior use and perceived knowledge of AI functioning.

### 3.2 MANOVA

The MANOVA results are reported in Table 3.

**Table 3:** MANOVA results.

**Variables**	**Men**		**Women**		***F*_(1, 332)_**	**η^2^*p***
	***M***	**SD**	***M***	**SD**		
1. AI anxiety	3.52	1.19	4.00	1.13	14.34^***^	0.04
2. Positive attitudes toward AI	3.40	0.84	2.97	0.66	27.16^***^	0.07
3. Prior use of AI	4.37	1.98	3.21	1.84	30.55^***^	0.08
4. Perceived knowledge of AI functioning	4.17	1.71	2.99	1.32	49.87^***^	0.13

^***^p < 0.001.

The MANOVA results revealed significant differences between groups (Wilk's Λ = 0.838, partial η^2^ = 0.162, *p* < 0.01). Specifically, men and women differed significantly on AI anxiety, positive attitude toward AI, prior use of AI, and perceived knowledge of AI functioning.

### 3.3 Effect of AI anxiety on AI attitudes and gender moderation

The results of the moderation model are reported in Table 4.

**Table 4:** Moderation model results.

**Effect**	***b***	**SE**	**95% CI**	
			**Lower**	**Upper**
Age	−0.00	0.00	−0.00	0.00
PAIU	0.12	0.02	0.07	0.16
PKAIF	−0.00	0.03	−0.06	0.04
AIA	−0.42	0.10	−0.61	−0.22
Gender	−0.75	0.25	−1.23	−0.26
AIA^*^gender	0.15	0.06	0.02	0.26

Dependent variable: positive attitudes toward AI. PAIU, prior use of AI; PKAIF, perceived knowledge of AI functioning; AIA, AI anxiety.

The model accounted for 32% of the variance in the criterion [*F*_(6, 327)_ = 25.481, *p* < 0.01]. Results revealed the main effect of AI anxiety on positive attitudes toward AI. In detail, higher levels of AI anxiety were positively related to lower levels of positive attitude toward AI. Furthermore, results showed a main effect of gender on positive attitude toward AI, with men reporting higher levels of positive attitudes toward AI than women as expected.

**Figure 1:**
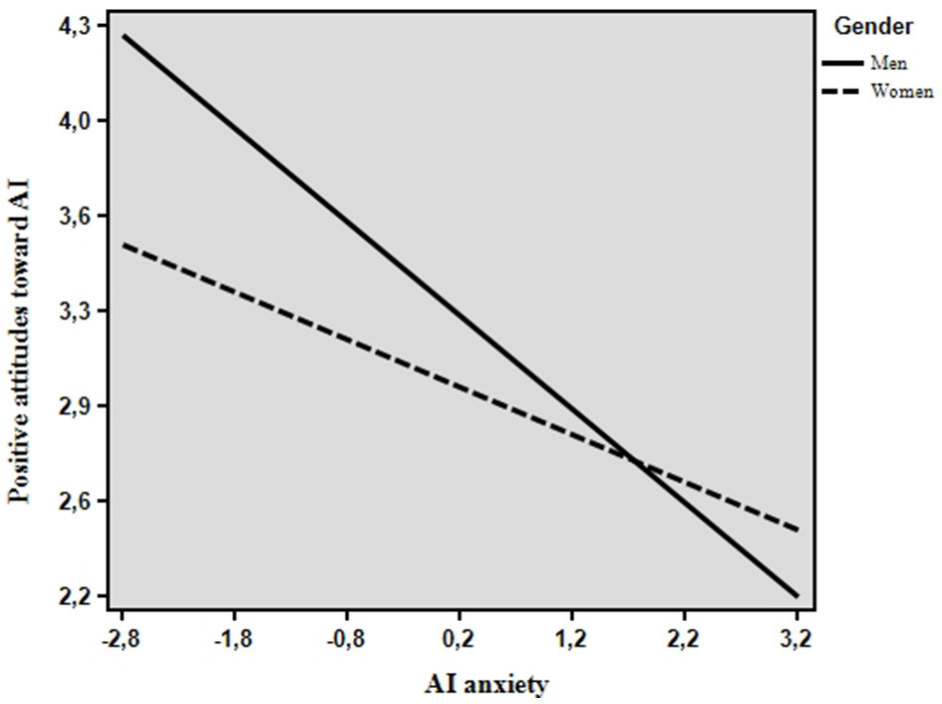
Moderation of gender in the relationship between AI anxiety and positive attitudes toward AI.

Finally, the interaction between AI anxiety and gender was significant. Specifically, simple slope analysis (Figure 1) showed that the negative relationship between AI anxiety and positive attitudes toward AI is slightly stronger for men (*b* = −0.27, SE = 0.05; 95% CI = −0.36, −0.18) than women (*b* = −0.13, SE = 0.04; 95% CI = −0.22, −0.04).

Graphically, it appears that higher levels of AI anxiety mainly affect men's attitudes toward AI compared to women. However, at higher levels of AI anxiety, gender differences are less evident. On the contrary, at lower levels of AI anxiety, women showed lower levels of positive attitudes toward AI than men.

Concerning the covariates included in the model, results revealed a significant positive effect of prior use of AI on positive attitudes toward AI. No significant direct effects were found for age and perceived knowledge of AI functioning.

## 4 Discussion

The present study aimed to identify gender differences in different dimensions that reflect AI adoption, such as AI anxiety, positive attitudes toward AI, perceived AI knowledge, and AI use. Moreover, we examined the relationship between AI anxiety and positive attitudes toward AI, taking into account the moderating role of gender. We hypothesized that women would report higher levels of AI anxiety and lower levels of positive attitudes toward AI, perceived AI knowledge, and AI use than men. We also hypothesized a negative relationship between AI anxiety and positive attitudes toward AI, with a significant moderation of gender.

Consistent with H1, women reported lower levels of positive attitudes toward AI, perceived AI knowledge, and AI use and higher levels of AI anxiety compared to men. These findings can be understood in light of the digital gender gap and gender-based socialization processes that shape women's interaction with technologies (Davaki, 2018). The higher levels of AI anxiety reported by women may be explained, on one side, by gender stereotypes that discourage women from delving into STEM fields and acquiring technological skills (Ertl et al., 2017). However, AI anxiety can be influenced not only by objective barriers but also by subjective feelings of inaccessibility and inadequacy, which can be highly amplified in contexts where women perceive a lack of role models and support (European Parliament, 2012). Moreover, women's lower exposure to new technologies like AI systems may increase their perceived complexity of AI functioning, leading them to perceive less knowledge about these systems and reducing their interaction with and their positive attitudes toward AI (Ofosu-Ampong, 2023). Consistently, previous studies have shown that women tend to perceive AI systems, such as robots, as less socially desirable than men (Schermerhorn et al., 2008; Kuo et al., 2009), considering these innovations to worsen face-to-face communication, lead to unneeded consumption behaviors, and increase unemployment (Méndez-Suárez et al., 2023). Additionally, our results highlighted that AI anxiety is significantly and negatively related to positive attitudes toward AI (H2) and that this relationship varies as a function of gender (H3). Like every other type of attitude, attitudes toward AI are shaped by emotions (Park and Woo, 2022). Consistent with previous evidence (e.g., Aggelidis and Chatzoglou, 2009; Chen and Tseng, 2012; Alrajawy et al., 2018; Kaya et al., 2024), anxiety is an affective response that can interfere with the formation of positive attitudes, influencing both perceived usefulness and behavioral intention, leading to a significant reduction of people's enthusiasm toward technological advancement. Our results are in line with and expand the Technology Acceptance Model (TAM; Davis, 1989). Indeed, our results show that emotional factors like anxiety might be directly associated with attitudes toward technological innovations.

Interestingly, our findings highlighted that gender significantly moderates this relationship. Specifically, our results show that higher levels of AI anxiety mainly affect men's attitudes toward AI compared to women, although gender differences are less evident at high levels of anxiety. Still, at low levels of AI anxiety, women report lower levels of positive attitudes toward AI than men. These findings can be again partially discussed in light of socio-cultural dynamics strictly related to the gender digital gap. Indeed, as previously shown by several research, women are often socialized to view technological domains as less congruent with their expected gender roles (Cuddy et al., 2008; Haines et al., 2016). As a result, women's attitudes toward AI may be less influenced by emotional states such as anxiety, as long-standing gendered socialization processes may mainly shape their skepticism toward AI. Thus, at low levels of anxiety, gender digital gap dynamics rise more strongly, leading women to display less positive attitudes than men. On the contrary, although higher levels of AI anxiety have a slightly stronger impact on men's attitudes, negative emotional experiences predominate for both genders, acting as a “gender differences leveler.”

### 4.1 Limitations and future research directions

In interpreting these findings, three main limitations should be acknowledged. Firstly, the cross-sectional nature of the study did not allow us to grasp the causality of the relationship between the variables. Future studies should adopt an experimental design. Secondly, our sample is mainly composed of Italians and young adults, limiting the generalizability of the results to other contexts and different ages. Thus, it might be relevant to replicate the study involving participants from various countries and different ages (i.e., adolescents and older people). Finally, we only adopted self-report measures. Future studies should also include the behavioral or physiological measures of anxiety while using AI, leading to further evidence that could strengthen these findings.

In addition to the directions for further research that would address specific limitations of the current study, our findings emphasize the need to adopt a multifactorial approach to the study of attitudes toward AI, considering both psychological—such as anxiety—and socio-demographic factors—such as gender. For example, it would be interesting to explore further whether and to what extent the gender differences found in the current study are related to different expectations of AI. For example, Generative AI might be perceived by women as “fake people,” whereas, for men, it might be seen as a cold machine that is useful for achieving their goals.

### 4.2 Policy implications

As Kong et al. (2020) stated, it is crucial to continue doing research in this direction, with the final aim of guiding policies. Our findings illustrate that women face barriers related to AI in ways that may be subtle yet highly consequential. The gender gap in STEM fields is not solely a matter of individual preferences or psychological factors such as anxiety; rather, it is deeply rooted in structural and sociocultural inequalities (Chavatzia, 2017; Cheryan et al., 2017). These inequalities shape early educational experiences, career aspirations, and professional opportunities, ultimately influencing attitudes toward AI and other technological advancements. While both men and women may experience apprehension regarding AI, psychological interventions aimed at reducing anxiety alone are insufficient to close the gender gap in technological engagement. Research suggests that broader systemic factors—such as stereotypes, lack of female representation, and limited access to resources—play a crucial role in shaping women's participation in STEM (Dasgupta, 2011). Addressing these structural disparities is therefore essential to fostering more inclusive technological environments.

One approach might be to increase the visibility of successful women in technology and AI-related fields. Studies on the role model effect suggest that exposure to women who are experts in STEM can help challenge gender stereotypes and increase women's sense of belonging in STEM disciplines (Dasgupta, 2011). Moreover, interventions at an early developmental stage are particularly promising. Research on educational inclusivity suggests that when girls encounter counter-stereotypical role models in childhood, they are more likely to develop an interest in STEM and persist in these fields later in life (Master et al., 2016). Policymakers and educators should, therefore, prioritize initiatives that promote diverse role models and inclusive educational programs. Strategies such as integrating gender-sensitive STEM curricula, fostering mentorship networks, and supporting women in leadership positions within the AI sector could be instrumental in reducing the gender digital divide. Additionally, ensuring equitable access to AI-related training and education can empower women to engage in technological innovation. By addressing these structural barriers, we can move beyond simply mitigating anxiety about AI to a more transformative approach that actively addresses gender disparities in technology. This shift could ensure that AI development and implementation reflect diverse perspectives, ultimately leading to more equitable and effective technological progress.

## 5 Conclusions

To the best of our knowledge, this is the first study that simultaneously investigated gender differences in different AI adoption indicators and assessed the relationship between AI anxiety and AI attitudes, also considering the moderating role of gender. Results showed gender differences as well as a moderating effect of gender on the relationship between AI anxiety and positive attitudes toward AI, with gender differences playing a stronger differentiating role at lower levels of anxiety. This study addresses the intersection of the critical topics of AI and gender equality and provides a foundation for future psychological research and technology and social inclusion policy.

## Data Availability

The raw data supporting the conclusions of this article will be made available by the authors, without undue reservation.
